# Tacks vs. sutures: a biomechanical analysis of sacral bony fixation methods for laparoscopic apical fixations in the porcine model

**DOI:** 10.1007/s00404-021-06343-w

**Published:** 2021-11-29

**Authors:** Alina Katharina Jansen, Sebastian Ludwig, Wolfram Malter, Axel Sauerwald, Jens Hachenberg, Caroline Pahmeyer, Kilian Wegmann, Claudia Rudroff, Leonidas Karapanos, Julia Radosa, Nadja Trageser, Christian Eichler

**Affiliations:** 1 Department of Gynecology and Obstetrics, Faculty of Medicine and University Hospital Cologne, University of Cologne, Kerpener Straße 62, 50931 Cologne, Germany; 2 Department of Gynecology and Obstetrics, St. Marien Hospital Düren, Düren, Germany; 3 Department of Gynacology and Obstetrics, Hannover Medical School, Hannover, Germany; 4 Faculty of Medicine and University Hospital Cologne, Department for Trauma, Hand and Elbow Surgery, University of Cologne, Cologne, Germany; 5 Department of General Surgery, Evangelisches Krankenhaus Köln-Weyertal, Cologne, Germany; 6 Department of Urology, Uro-Oncology, Robot-Assisted and Reconstructive Surgery, Faculty of Medicine and University Hospital Cologne, University of Cologne, Cologne, Germany; 7 Department for Gynecology, Obstetrics and Reproductive Medicine, Saarland University Hospital, Homburg, Germany; 8 Breast Cancer Center, St. Franziskus- Hospital Münster, 48145 Münster, Germany

**Keywords:** Pelvic organ prolapse, Urogynaecological surgery, Cervicosacropexy, Vaginosacropexy, Uterosacral ligaments, Polyvinylidene-fluoride

## Abstract

**Purpose:**

There is a novel surgical procedure, called cervicosacropexy (CESA) and vaginosacropexy (VASA) to treat pelvic organ prolapse and a concomitant urgency and mixed urinary incontinence. As there is little experience with the tapes so far and literature is scanty, the aim of this study was to investigate biomechanical properties for the fixation of the PVDF-tapes with three different fixation methods in context of apical fixations.

**Methods:**

Evaluation was performed on porcine, fresh cadaver sacral spines. A total of 40 trials, divided into 4 subgroups, was performed on the anterior longitudinal ligament. Recorded biomechanical properties were displacement at failure, maximum load and stiffness in terms of the primary endpoints. The failure mode was a secondary endpoint. Group 4 was a reference group to compare single sutures on porcine tissue with those on human tissue. Biomechanical parameters for single sutures on the human anterior longitudinal ligament were evaluated in a previous work by Hachenberg et al.

**Results:**

The maximum load for group 1 (two single sutures) was 65 ± 12 N, for group 2 (three titanium tacks arranged in a row) it was 25 ± 10 N and for group 3 (three titanium tacks arranged in a triangle) it was 38 ± 12 N. There was a significant difference between all three groups. The most common failure mode was a “mesh failure” in 9/10 trials for groups 1–3.

**Conclusion:**

The PVDF-tape fixation with two single sutures endures 2.6 times more load than titanium tacks arranged in a row and 1.7 times more load than titanium tacks arranged in a triangle. The presacral fixation with titanium tacks reduced surgical time compared to the fixation with sutures, nevertheless sutures represent the significantly stronger and cheaper fixation method.

## Introduction

Pelvic organ prolapse (POP) describes the descent of one or more structures in the small pelvis. The bladder, uterus or vagina may be affected and consequently the anatomical position is not given anymore [1]. There are multifactorial reasons; however, an increasing age, a high body-mass index and previous vaginal child births are the main risk factors for pelvic organ prolapse [2]. Zargham et al. showed that the parameter age is significantly related to an increasing POP grade, no matter which type of prolapse, because of the increasing atrophy of pelvic structures, such as muscles and ligaments and an increasing connective tissue disorder [3]. In addition to that, urinary incontinence is an increasing problem, also becoming more important in the elderly population [3, 4]. The prevalence depends on the study, nevertheless, Thom et al. detected a prevalence of urinary incontinence about 17–55% for older women [4]. Interestingly, with an increasing age, the percentage of women with stress incontinence decreases, whereas urge and mixed incontinence become more frequent [3, 4].

Pelvic organ prolapse and urinary incontinence interact and coexist [5]. Accordingly, Buchsbaum et al. described that 60% of the patients with POP were also suffering from incontinence and in 40% of the patients with incontinence, there was a coexisting prolapse as well [6]. According to Ulmsten, Petros and DeLancey, stress and urgency urinary incontinence result from the laxity of the anterior vaginal wall [7, 8]. Defects of the holding apparatus of the uterus and vagina, and thus especially of the uterosacral ligaments (USL), lead to laxity of the anterior vaginal wall and thus cause prolapse and also urinary incontinence. To restore the anatomy of the holding apparatus, Jäger et al. decided to repair the USL in patients with an apical descent (either of the uterus or vaginal vault) and a concomitant urinary incontinence [9, 10]. This novel surgical technique, called cervicosacropexy (CESA) and vaginosacropexy (VASA), aimed to restore the original anatomy by replacing the USL bilaterally with a minimum of alloplastic material. The operation was “standardized” by using polyvinylidene fluoride (PVDF) tapes of a defined identical length and width for all women [9, 10]. Polyvinylidene fluoride (PVDF) meshes were shown as a possible alternative to commonly used polypropylene (PP) meshes, as PVDF goes along with a reduction of mesh-related side effects. PVDF shows a great biostability, a lowered bending stiffness and a minimum tissue reaction with low inflammation parameters [11, 12].

Due to the advantages of laparoscopy, the CESA and VASA surgical technique was adapted into laparoscopic approaches [13–15].

**Fig. 1:**
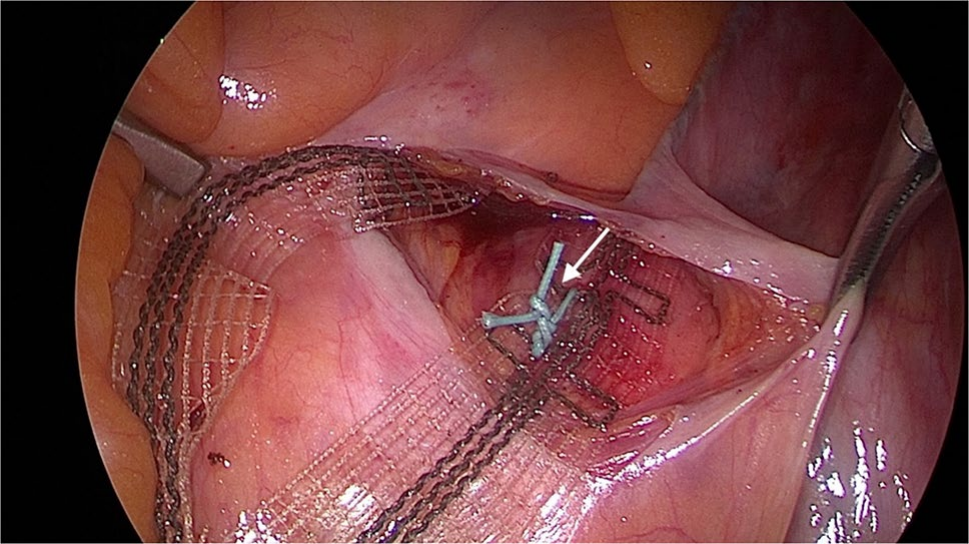
Intraoperative posterior fixation. Posterior fixation of the PVDF ligament-replacement structure (DynaMesh CESA, FEG Textiltechnik mbH Aachen, Germany) with nonabsorbable sutures (white arrow), PremiCron (HR26s, braided, coated, non-absorbable sutures, Braun Surgical, S.A. Rubi. Spain). Note that only one suture is shown in this figure

Since there is always the aim to reduce hospitalization and improve reconvalescence, Rexhepi et al. performed laparoscopic cervicosacropexy (laCESA) and laparoscopic vaginosacropexy (laVASA). By the restoration of prolapse, urinary continence could be reestablished in 64% of patients with mixed urinary incontinence and in 69% of the patients with urgency incontinence. In addition to those excellent results in success rate, Rexhepi and et al. reported a possible operation time of less than 1 h, depending on the patient´s constitution and other factors, and a short hospitalization of 3 days [13]. One main difference to the open abdominal surgical technique described by Jäger et al. was the posterior fixation at the sacral vertebra. In abdominal CESA or VASA, two non-absorbable sutures for the presacral tape fixation were used [10], whereas in laparoscopic CESA or VASA the fixation device changed and three titanium tacks were used instead of two sutures [13]. The right anatomical position for the sacral fixation at the pre-vertebral fascia is at the level of S1 and S2 [10]. Figure 1 shows the posterior fixation of the PVDF ligament-replacement structure with nonabsorbable sutures.

Since laparoscopic suturing prolongs the operation time in laCESA or laVASA compared to the use of the fixation device with titanium tacks, the question arose if there are comparable results between the sutures, which are used as standard by Jäger et al. [10] and other study groups [16, 17] and the ProTack™ tacks and if one fixation method is superior. Moreover, the aim of this study was to find out, if there is a difference depending on how the tacks are arranged. In group 2, the titanium tacks are arranged in a row, whereas in group 3 the tacks are arranged in a triangle. Any deviation from gold standard should, however, be first supported with biomechanical data.

Therefore, the aim of the study was to explore and compare the biomechanical properties of the interrupted sutures (gold standard) and both ProTack™ groups, each in combination with the PVDF-tape and to figure out the limiting factor, i.e., failure mode for the different fixation methods (mesh, tissue, fixation device). A further aim was to experience, if the biomechanical properties obtained on porcine tissue are comparable to those on human tissue.

## Methods

The evaluation of different fixation methods was performed on non-embalmed, fresh, unfrozen porcine cadaver sacral spines. An experienced urogynaecological surgeon prepared the prevertebral fascia/ anterior longitudinal ligament. 40 halved porcine spines were used for the experiments. All cadavers were procured in an appropriate quality from a slaughterhouse in Wachtendonk, Germany and used for the experiments on the same day. A total of 40 trials were performed. Each cadaver was only used in one trial. 4 types of trials were conducted. Group 1 (*n* = 10) used two single sutures to attach the PVDF-tape on the prevertebral fascia. Group 2 (*n* = 10) evaluated the PVDF-tape fixation with three titanium tacks arranged in a row (ProTack™, Covidien, Mansfield, MA), group 3 (*n* = 10) evaluated the PVDF-tape fixation with three titanium tacks arranged in a triangle (ProTack™, Covidien, Mansfield, MA) and group 4 (*n* = 10) evaluated a single suture without any mesh on the anterior longitudinal ligament.

The first three groups evaluated and compared the different fixation devices used with the PVDF-tape, whereas the fourth group was a reference group to compare single sutures on porcine tissue with those on human tissue. In a previous work Hachenberg et al. evaluated a single suture orthogonally placed to the human anterior longitudinal ligament and captured data for the biomechanical parameters displacement at failure (mm), maximum load (N) and stiffness (N/mm) [18].

**Fig. 2:**
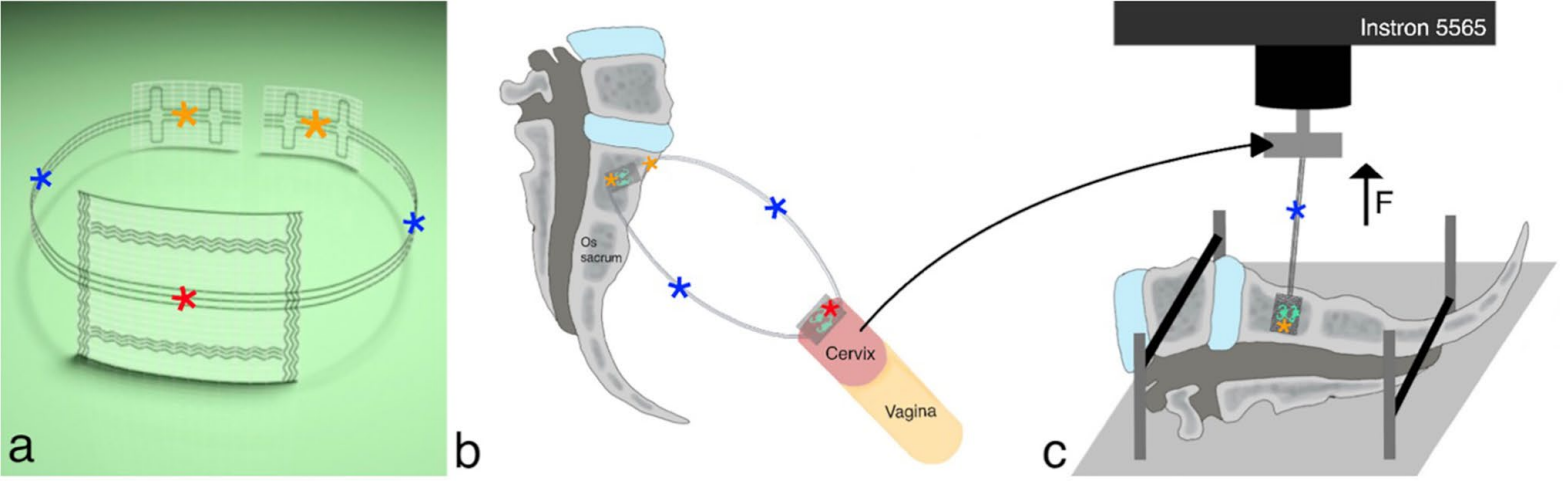
Schematic view of the PVDF tape in the small pelvis and in the testing frame. **a** Specially designed polyvinylidene fluoride (PVDF) tape (shown: DynaMesh CESA, FEG Textiltechnik mbH Aachen, Germany). The red asterisk shows the central part of the tape for cervical fixation. The two blue asterisks show the arms of the PVDF-tape, which are intended for uterosacral ligament replacement on both sides of the small pelvis. The two orange asterisks represent the fixation surface for posterior fixation to the sacral vertebra on the left and right side of the small pelvis at the level of S1/S2. **b** Schematic view of the above described PVDF- tape in the small pelvis. **c** Schematic view of the testing setup used for the in vitro tests in groups 1–3. The black arrow shows that the cervix is replaced by the Instron 5565^®^ and the central part of the PVDF-tape is fixed in the test frame. The Instron 5565^®^ exerts a force (F) from above to evaluate the biomechanical parameters maximum load (N), displacement at failure (mm) and stiffness (mm/N) for the sacral tape fixation. The orange asterisk shows the posterior fixation of the PVDF-tape, either with two single sutures, PremiCron (HR26s, braided, coated, non-absorbable sutures, Braun Surgical, S.A. Rubi. Spain) or with three tacks (ProTack, Auto Suture 5 mm, Covidien, Mansfield, MA, USA)

The utilised mesh, previously described as tape, has a defined shape and is knitted from non-absorbable, biostable polyvinylidene fluoride monofilaments (DynaMesh^®^ CESA, FEG Textiltechnik mbH Company, Aachen, Germany). This PVDF-tape consists of a central part (3 × 4 cm) and two 8.8 cm long and 0.4 cm wide arms (intended for uterosacral ligament replacement). At the ends of both arms there is a 1 × 2 cm fixation surface for fixation to the sacral vertebra (Fig. 2a). A polyester, braided, coated, non-absorbable PremiCron^®^ suture 1, HR26s needle, 75 cm green filament (Braun Surgical, S.A. Rubi. Spain) was used in group 1 and in group 4.

The analysis was performed on an Instron 5565^®^ test frame using the Bluehill 2 Software^®^. All tests evaluated the individual fixation method and started with a preload of 1 N. A schematic view of the above described PVDF-tape location in the small pelvis, as well as the transfer to the in vitro tests for groups 1–3 is shown in Fig. 2b, c.

The analysis resulted in three primary endpoints. Maximum Load (N) and displacement at failure (mm) were directly measured by using the Instron 5565^®^. According to that, the third parameter stiffness (N/mm) was calculated by using the previous results. Maximum load was defined as the highest achieved force in Newton (N) that the fixation could withstand before it loosened. The displacement at failure was defined as the elongation of the construct in millimetre (mm) up to the point, where the maximum load was reached. Stiffness was calculated as the slope of the linear elastic region and describes the elongation due to the acting force in Newton (N).

Secondary endpoint was the evaluation of the failure mode for groups 1–3.

### Procedure

**Fig. 3:**
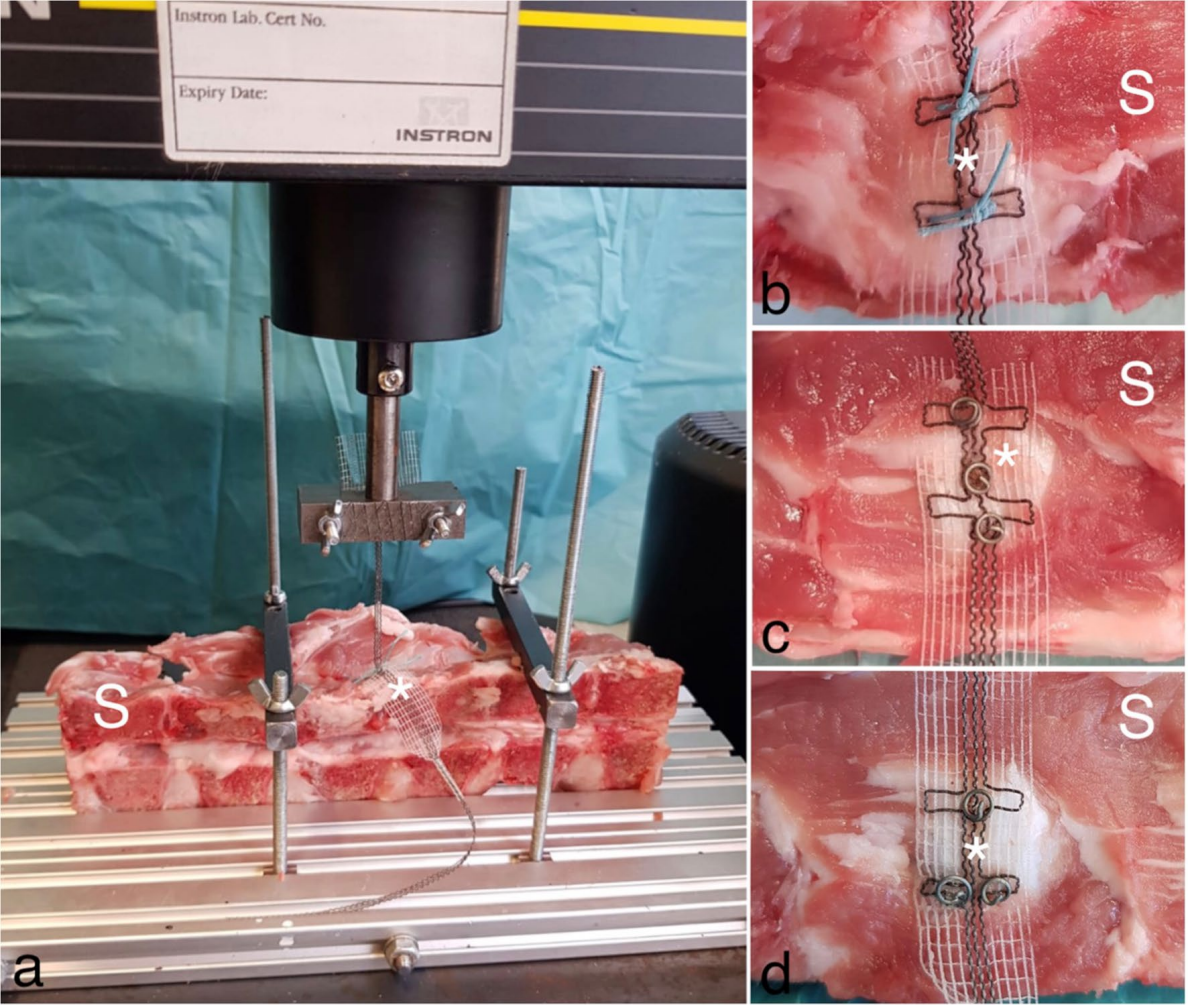
Testing frame and evaluated fixation methods. Shown are representative images of the complete testing setup with the porcine spines (S). The white asterisks show the parts of the PVDF-tape (DynaMesh CESA, FEG Textiltechnik mbH Aachen, Germany) for sacral fixation at the prevertebral fascia at the level of S1/S2. **a** Shown is the complete testing setup during the dynamic testing. **b** Mesh fixation to the prevertebral fascia in group 1 with two single sutures. Group 1 used PremiCron (HR26s, braided, coated, non-absorbable sutures, Braun Surgical, S.A. Rubi. Spain). **c** Mesh fixation to the prevertebral fascia in group 2 with three titanium tacks arranged in a row (ProTack, Auto Suture 5 mm, Covidien, Mansfield, MA, USA). **d** Mesh fixation to the prevertebral fascia in group 3 with three titanium tacks arranged in a triangle (ProTack, Auto Suture 5 mm, Covidien, Mansfield, MA, USA)

The test setup is shown in Fig. 3. The cadaver spines were prepared and cut in a manner they could easily be placed in the test frame. Cadavers were chilled, but not frozen. Figure 3b shows the tape fixation with two single sutures, which are orthogonally placed to the tape in the direction of the fiber. Figure 3c shows the tape fixation with three titanium tacks placed in a row and Fig. 3d shows the tape fixation with three titanium tacks placed in a triangle. All three fixation methods led to an adequate tape surface interaction. The USL replacement structures of the tape were used to fix it in the metal clamp of the Instron test frame. The length between the suture or the titanium tacks and the metal clamp was the same for all trials. Therefore, equal and comparable conditions were given. All trials were performed until mesh, tissue or fixation device failure occurred.

### Statistics

Statistical analysis was performed using the VassarStats^®^ (Vassar College, Poughkeepsie, NY, USA) statistics program. ANOVA analysis was used to evaluate significances when appropriate.

## Results

A total of 40 trials, subdivided in four groups, were conducted between June and November 2020 at the University of Cologne, Department of Anatomy, Cologne, Germany.

**Fig. 4:**
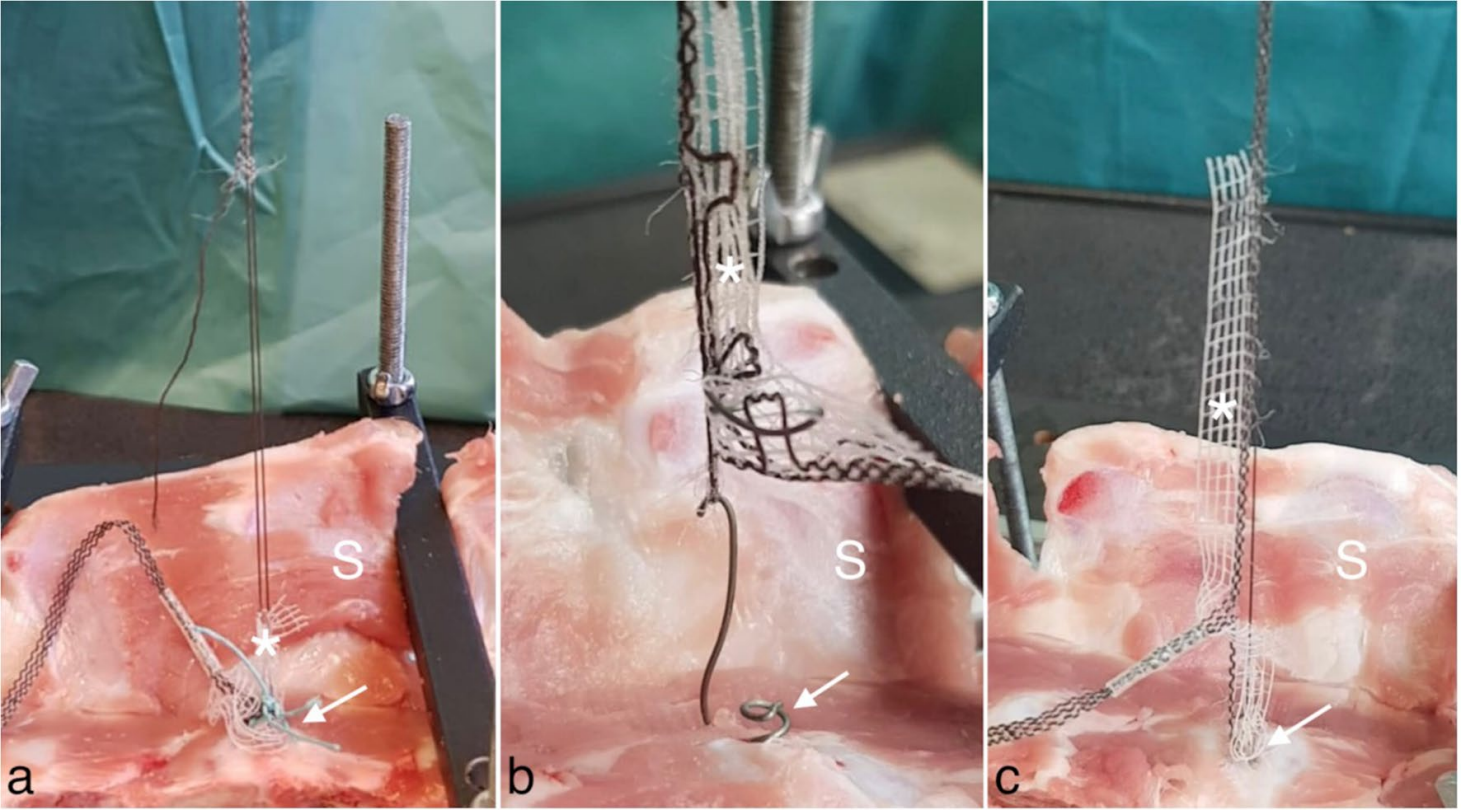
Failure Mode for groups 1 and 2. Shown are the different failure modes for group 1 (Suture Spine), which used PremiCron (HR26s, braided, coated, non-absorbable sutures, Braun Surgical, S.A. Rubi. Spain) and group 2 (ProTack™ Spine in a row), which used a fixation device (ProTack, Auto Suture 5 mm, Covidien, Mansfield, MA, USA) for fixation of the PVDF (polyvinylidene fluoride) tape (DynaMesh CESA, FEG Textiltechnik mbH Aachen, Germany) to the porcine sacral spine (S). The white arrow shows the prevertebral fixation either with sutures or tacks. The white asterisks show the PVDF-tape. **a** Shown is the mesh failure for group 1. **b** Shown is the fixation device failure for group 2. **c** Shown is the mesh failure for group 2

A summary of the results is given in Tables 1 and 2. Group 1 represents the gold standard fixation with two non-absorbable sutures and the PVDF-tape. On the contrary, group 2 and group 3 represent the fixation with three titanium tacks (ProTack™), once arranged in a row and once arranged in a triangle, and the PVDF-tape. In all three groups, most frequently, the tape showed to be the limiting factor. In group 1, one trial had to be terminated because of a tissue failure, whereby the anterior longitudinal ligament ruptured, which caused the failure of the tape fixation. Figure 4a illustrates the failure mode “mesh failure” in group 1. The most common location for failure was the arms (thin part) of the PVDF-tape, namely, the part that replaces the USL.

**Table 1:** Overall results for the three evaluated groups performed on the porcine sacral spines

Evaluated entity	Number tested	Maximum load	Displacement at failure	Stiffness	Failure mode
Total trials (*n* = 20)	*n*	N	mm	N/mm	
Group 1 (suture spine)	10	65 (± 12)	36 (± 3)	2.43 (± 0.50)	Mesh failure 9/10, tissue failure 1/10
Group 2 (ProTack™ Spine in a row)	10	25 (± 10)	29 (± 14)	1.12 (± 0.22)	Mesh failure 9/10, fixation device failure 1/10
Group 3 (ProTack™ Spine in a triangle)	10	38 (± 12)	44 (± 11)	1.01 (± 0.15)	Mesh failure 9/10, fixation device failure 1/10

Group 1 (suture Spine) used PremiCron (HR26s, braided, coated, non-absorbable sutures, Braun Surgical, S.A. Rubi. Spain), group 2 (ProTack™ spine in a row) and group 3 (ProTack™ Spine in a triangle) used a fixation device (ProTack, Auto Suture 5 mm, Covidien, Mansfield, MA, USA) for fixation of the PVDF (polyvinylidene fluoride) meshes (DynaMesh CESA, FEG Textiltechnik mbH Aachen, Germany)

N, Newton; mm, millimeter

**Table 2:** Comparison of the results for group 4, performed on porcine sacral spines with the data collected on human sacral spines

Evaluated entity	Number tested	Maximum load	Displacement at failure	Stiffness
Total trials (*n* = 18)	*n*	*N*	mm	N/mm
Single suture on porcine sacral spine	10	89 (± 10)	16 (± 2)	5.31 (± 0.97)
Single suture on porcine sacral spine^a^	8	61 (± 29)	29 (± 6)	2.99 (± 0.86)
*p* values		0.0306	0.0005	< 0.0001

N, Newton; mm, millimeter

^a^Those data were published by Hachenberg et al. [18]

In group 2, in one trial the fixation device was the limiting factor (Fig. 4b). In this case one of the titanium tacks bended up and the other two broke out of the ligament completely. In the remaining nine trials, the mesh was the limiting factor. The mesh failure for group 2 is shown in Fig. 4c. In two trials the location for failure was, comparable to the sutures, the thin part of the PVDF-tape. In seven trials the mesh snapped out of the titanium tacks and was thus split into two parts. In group 3, there occurred a mesh failure in 9 of 10 trials and a fixation device failure in one trial.

The maximum load was 65 ± 12 N for group 1, 25 ± 10 N for group 2 and 38 ± 12 N for group 3. There was a significant difference in the maximum load between groups 1 and 2 (*p* < 0.01), as well as between groups 2 and 3 (*p* = 0.023) and between group 1 and group 3 (*p* < 0.01). Regarding the parameter displacement at failure, there is no significant difference between group 1 (36 ± 3 mm) and group 2 (29 ± 14 mm), nor between group 1 and group 3 (44 ± 11 mm), but there is a significant difference between group 2 and group 3 (*p* < 0.01). Surveying the third parameter, stiffness, there are values of 2.43 ± 0.50 for group 1, 1.12 ± 0.22 for group 2 and 1.01 ± 0.15 for group 3. There is a significant difference between group 1 and group 2 (*p* < 0.01) and between group 1 and group 3 (*p* < 0.01), whereas there is a non-significance between group 2 and group 3.

Group 4 was used as a control group and represents the single suture without any mesh on the anterior longitudinal ligament. All trials in group 4 were limited by suture tearing. A maximum load of 89 ± 10 N could be achieved for porcine tissue, whereas Hachenberg et al. [18] figured out a maximum load of 61 ± 29 N for the trials with a single suture orthogonally placed to the human anterior longitudinal ligament (*p* = 0.0306). Comparing those data, there was a slight difference, but the data remain at the same order of magnitude. The same applies to the parameter displacement at failure which was 16 ± 2 mm in group 4 and 29 ± 6 mm for human tissue (*p* = 0.0005) and the recorded parameter stiffness with values of 5.31 ± 0.97 mm/N for porcine tissue and 2.99 ± 0.86 mm/N for human fiber [18] (*p* < 0.0001).

## Discussion

The previously described CESA or VASA surgical technique is a standardized operation with a defined tape localization, defined fixation sides and identical lengths of the PVDF-tape for all patients [10]. On the one hand this standardization offers a big advantage and enables comparability and reproducibility among different centres [10], but on the other hand there is hardly any possibility to reduce surgical time, since all steps are necessary and none can be omitted.

To keep the surgical time as short as possible, and due to the fact, that laparoscopic suturing is a time-consuming task [19], it is the only possibility to optimize the individual steps.

According to our knowledge, there has thus far been three biomechanical analysis performed on human female cadaver pelvices. The first biomechanical analysis, conducted by Sauerwald et al. [19], compared the mesh fixation with a single suture on the ilio-pectineal ligament in pectopexy with the gold standard, a continuous approach, and figured out, that the use of a single suture is not inferior to a continuous approach. The second study, also conducted by Sauerwald et al. [20] examined the time until functional stability in pectopexy by means of cyclic testing and detected that functional stability is achieved after 19.1 cycles of a load exposure of below 25 N. Therefore, patients do not have to fear a global fixation failure. The third biomechanical analysis found in the current literature performed by Hachenberg et al. [18] is already mentioned above. This study, performed on human cadaver pelvices, showed that an orthogonally placed suture is superior to an in-line suture at the sacrospinal ligament. Moreover, it is shown, that a continuous suture is superior to a single suture at the anterior longitudinal ligament. Comparing those results with the data collected in group 4, the data remain at the same order of magnitude. Especially considering that Hachenberg et al. [18] performed the tests on patients with the average age of 75 years, whereas the porcine cadaver pelvices are from young pigs with a tighter tissue. Therefore, the results obtained in groups 1, 2 and 3 are transferable to human sacral spines.

In terms of the maximum load the collected data in groups 1 and 2, as well in group 1 and group 3, showed a significant difference between the respective fixation devices. Therefore, it can be concluded, that the tape fixation with two single sutures endures 2.6 times more load than the fixation with three tacks in a row and 1.7 times more load than the fixation with three tacks in a triangle. Comparing the results obtained in groups 2 and 3, in which the same fixation device was used, it could be seen a difference depending on how the tacks are arranged. Three tacks arranged in a triangle (group 3) supported 1.5 times the load that was supported in group 2.

In group 2, the tacks are arranged in a line one behind each other and thereon in 7 of 10 trials the mesh was divided into two parts. In addition, in group 3, those parts of the mesh, where the titanium tacks are attached were the predilection points. A possible explanation for the higher maximum load in group 3 is, that the load is evenly distributed on three fixation points, that are arranged in a triangle formation, so that the mesh is fixed wider and there is a reduced load at the individual fixation points.

The triangle formation represents more the physiological forces acting on the mesh, depending on which movement the patient is performing. If the tacks are arranged in a row, there is only one force vector acting on the fixation.

In a previous study, Formijne Jonkers et al. [21] compared three fixation techniques on the promontory in laparoscopic ventral rectopexy (LVR) and used a polypropylene mesh on a porcine spinal column. Comparable to the current study, the disruption force (DF), which corresponds to the parameter maximum load, was evaluated for the fixation with the ProTack™ tacks and sutures. Comparable to the trials in group 3, the three tacks were arranged in a triangle. Formijne Jonkers et al. [21] found data of 58 N for the three ProTack™ tacks and 55 N for the two stitches. There was no significant difference between the tacks and the stitches with regard to the strength of the mesh attachment [21]. Those results differ from the current study, as the superiority of the two single sutures could be shown in group 1. A possible explanation could be the different surgical technique and the use of a different mesh. To withstand a higher load in groups 1–3 of the current study, it would be necessary to repeat the trials with a more stable mesh.

Moreover, the data collected by Sauerwald and Eichler et al. [19] can be used for comparison. In their study, a maximum load of 35 ± 12 N was shown for the group, that examined the interrupted suture with the mesh on the ileo-pectinal ligament. Compared to the results for group 1 with a maximum load of 65 ± 12 N, there is a difference of 30 N, whereas the standard deviation is the same. Since the same fixation method was performed and since the mesh was the limiting factor in all trials, the significant difference in maximum load could be explained by the properties of the different meshes. Another explanation would be that a greater stability was achieved through the use of the second single suture and the mesh is thus better fixed and endures a higher load.

In group 3, the maximum load for the fixation with three titanium tacks was 38 ± 12 N. Even if there is a significant inferiority to the fixation with two single sutures, literature describes the successful use of three titanium tacks for the sacral fixation in clinical studies [13, 22]. In their clinical study, Rexhepi and Ludwig et al. [13] included 120 patients with urinary incontinence and performed laCESA or laVASA with three titanium tacks as sacral fixation. A relapse of apical prolapse could be shown after surgery because of the insufficient use of fast absorbable sutures at the cervix, but it did not occur any case of mesh erosion or reoperation because of an insufficient sacral fixation.

In a further study, Ludwig et al. [22] performed laCESA on a patient and also used the titanium tacks for the sacral fixation. Neither after surgery, nor 1 year postoperative, any prolapse could be detected. For evaluation of the maximum load, it is important to consider, that halved sacral spines were used and therefore a one-sided fixation was evaluated. One could assume twice the stability for a both-sided fixation.

In addition to that, in an in vivo-setting, the final surgical mesh stabilization is reached through wound healing and the mesh ingrowth. Thus, it can be assumed that all three groups can endure more load in vivo than in the described in vitro setting.

Based on the intraoperative measurements of the coauthor L.S., it takes in average 90 s to place one interrupted suture in laparoscopy, whereas it takes only 20 s to set three titanium tacks. In total for a both-sided presacral fixation with two single sutures on each side, it would occupy 6 min of surgical time. In contrast, for the both-sided fixation with three titanium tacks on each side it would take 40 s, which is 9 times faster than laparoscopic suturing. Considering the costs of the different fixation devices, sutures cost about 10 euro for 1 piece, whereas the ProTack™ fixation device costs about 300 Euro for one fixation device with 30 tacks, that is for a single use.

For the parameter displacement at failure, there were values of 36 ± 3 mm for group 1, 29 ± 14 mm for group 2 and 44 ± 11 mm for group 3. Comparing those results to current literature, comparable values are obtained. To reduce the surgical mesh placement time in laparoscopic surgery, Zimkowski et al. [23] compared the strain to failure for modified and unmodified surgical meshes and received a strain to failure of 21.92 ± 3.76 mm for a PET mesh, which was the control group. In the above-mentioned study, conducted by Sauerwald et al. [19], similar values for displacement at failure of 37 ± 12 mm were shown. From a clinical point of view, a maximum of 37 mm of displacement seems adequate [19]. This statement is also true for the evaluated values for displacement at failure obtained in groups 1–3.

The last parameter, stiffness, describes the resistance to the elastic deformation by an acting force. A significant difference for stiffness could be detected between groups 1 and 2, as well as between group 1 and group 3, whereby the stiffness was significantly higher in group 1 (2.43 ± 0.50) in contrast to group 2 (1.12 ± 0.22) and group 3 (1.01 ± 0.15). The PVDF-tape fixation with sutures was significantly stiffer than the fixation with titanium tacks. Between the two tack groups, there was a nonsignificant difference.

To sum up, the biomechanical analysis of different fixation methods (titanium tacks, ProTack™ and sutures) for the fixation of PVDF-tapes at the sacral vertebra yielded the following results:

Two interrupted sutures (group 1) in combination with the PVDF-tape supported a load up to 65 N, a displacement at failure of 36 mm and a stiffness of 2.43 mm/N, whereby the ProTack™ tacks, arranged in a row (group 2), supported a maximum load of 25 N, a displacement at failure of 29 mm and a stiffness of 1.12 mm/N. The ProTack™ tacks, arranged in a triangle (group 3), supported a maximum load of 38 N, a displacement at failure of 44 mm and a stiffness of 1.01 mm/N. To address the most common limiting factor, i.e., failure mode, there was a mesh failure for all three groups (in 9/10 trials for all groups). In 1/10 trials for group 1 occurred a tissue failure, in 1/10 trials for groups 2 and 3, there was a fixation device failure. To answer the question, if the biomechanical properties obtained on porcine tissue are comparable to those on human tissue, the results of group 4 were compared to the data collected on human porcine spines by Hachenberg et al. There is a slight difference, but the data remain at the same order of magnitude and could therefore be transferred to human tissue.

## Conclusion

Given the data above, it can be concluded that the PVDF-tape fixation with two single sutures in apical fixation is significantly superior to the fixation with three titanium tacks in terms of the maximum load (N) as sutures endures 2.6 times more load that is supported by titanium tacks in a row and 1.7 times more load than titanium tacks arranged in a triangle. Comparing both tack groups, the tack formation in a triangle (group 3) withstands 1.5 times more load than the tack formation in a row (group 2). This is an important and helpful finding for everyday clinical practice, as it is possible to achieve a higher load in the same operation time just through a different tack arrangement. Thus, it can be concluded that intraoperative, the ProTack™ tacks should preferentially be arranged in a triangle instead of a row to withstand a higher load.

The presacral fixation with three titanium tacks, whether in a row or in a triangle, reduces surgical time compared to the fixation with sutures. Nevertheless, the fixation with sutures represents the significantly stronger and cheaper fixation method.

As literature is scanty, the results of this study provide first insights on the comparison of different fixation methods used with the PVDF-tapes. There is the aim to achieve a standardization in this point as well, so the next steps in research would be to conduct further and larger studies with different fixation devices, as well as with different arrangement and number of fixation points to attach the PVDF-tapes sufficiently.

## Data Availability

The datasets generated and analyzed during the current study are available in the OSF repository, https://osf.io/ufctq/.

## Code availability

https://doi.org/10.17605/OSF.IO/UFCTQ.
